# Nitric oxide as a surgical adjuvant

**DOI:** 10.4155/fso.15.56

**Published:** 2015-08-01

**Authors:** Aimee Krausz, Adam J Friedman

**Affiliations:** 1 Department of Medicine (Division of Dermatology), Albert Einstein College of Medicine, Bronx, NY 10467, USA; 2 Department of Physiology & Biophysics, Albert Einstein College of Medicine, Bronx, NY 10461, USA; 3 George Washington School of Medicine & Health Sciences, WA, DC 20052, USA

**Keywords:** blood-contacting devices, ischemia reperfusion injury, wound healing

## Abstract

Advances in surgical technology have allowed for previously unconsidered therapeutic interventions. However, the complexity and invasiveness of surgical procedures are not without adverse consequences. Nitric oxide's fundamental role in a host of physiological processes, including angiogenesis, wound and bone healing, thromboresistance, smooth muscle relaxation and inflammation makes it a significant player in accelerating wound healing and mitigating the inflammation of ischemia reperfusion injury common to surgical procedures. In addition, the therapeutic properties of NO have been harnessed for the prophylactic treatment of implant infection and graft failure. In this article, we will discuss the mechanism by which NO mediates these processes, and its perioperative translational applications.

**Figure 1.**
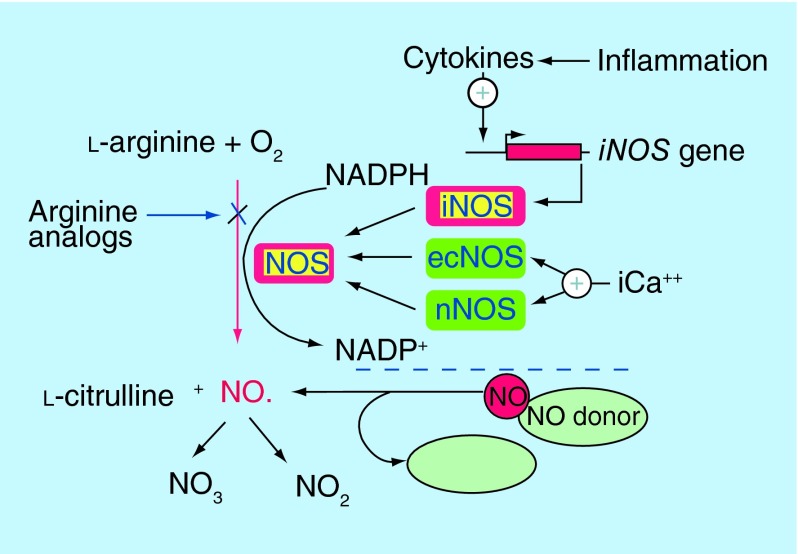
**Nitric oxide is synthesized from l-arginine and molecular oxygen by the NOS group of enzymes.** Inflammatory cytokines (IL-1, TNF-α) upregulated in response to tissue damage induce expression of the inducible *NOS* gene, leading to a more sustained release of NO. Endothelial NOS and neuronal NOS are constitutively expressed and regulated by calcium fluctuations. Reprinted with permission from [9] © John Wiley and Sons (2001).

**Figure 2.**
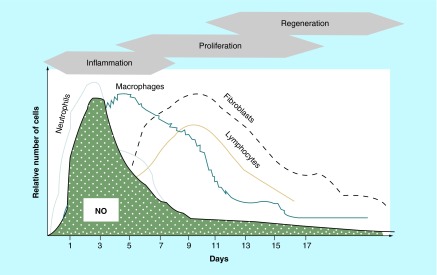
**Macrophage-induced generation of nitric oxide.** Inflammatory cells, primarily macrophages, are responsible for the largest generation of NO in wounds. This corresponds to the early peak of NO in the healing cascade. Reprinted with permission from [3] © Elsevier (2002).

**Figure 3.**
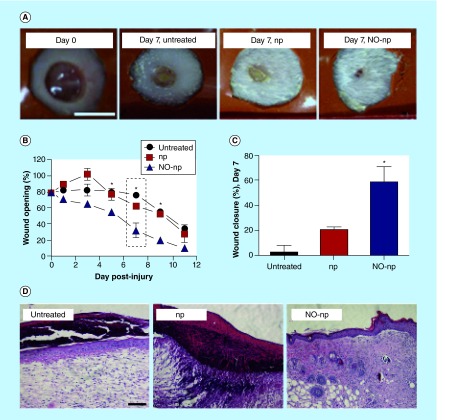
**Nitric oxide-nanoparticles enhance wound healing *in vivo*.** **(A)** Wounds of BALB/c mice at day 7. **(B)** Wound healing curve. **(C)** Wound closure percentage of BALB/c mice skin lesions at day 7 relative to the initial 5 mm wound. **(D)** Histological analysis of untreated, control-np and NO-np treated BALB/c mice at day 7. np: Nanoparticles. Reprinted with permission from [16] © American Society for Investigative Pathology (2012).

**Figure 4.**
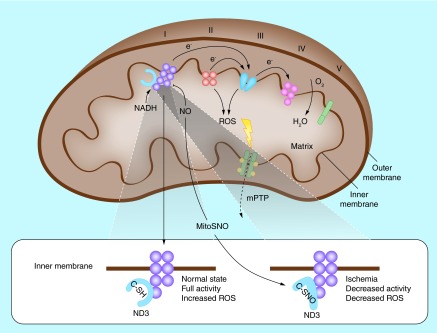
**In mitochondria, electrons move down the electron transport chain and are transferred to O_2_ at complex IV.** When oxygenation is normal, complex I activity is high because a cysteine residue on its ND3 subunit (C-SH) is protected from modification (bottom left). During ischemia, electrons (e^–^) accumulate along the ETC because O_2_ is absent. Reoxygenation then causes a burst of ROS generation from multiple sites, resulting in lethal activation of the mitochondrial permeability transition pore. ETC: Electron transport chain; ROS: Reactive oxygen species. Reprinted by permission from [24] © Macmillan Publishers Ltd, Nature Medicine (2013).

**Figure 5.**
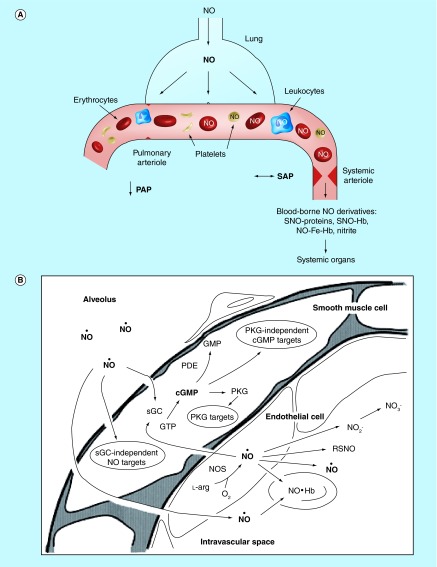
**Inhaled NO is a selective-pulmonary vasodilator with actions on the systemic vasculature.** A schematic of an alveolar-capillary unit is presented highlighting the ability of inhaled NO to dilate pulmonary arterioles and reduce PAP. Although inhaled NO does not dilate systemic arterioles or alter SAP, inhaled NO does have systemic effects mediated by circulating cells exposed to NO in the lungs and blood-borne NO derivatives. PAP: Pulmonary artery pressure; SAP: Systemic arterial pressure. **(A)** Reprinted with permission from [29] © European Society of Cardiology (2007). **(B)** Reprinted with permission from [27] © American Heart Association (2004).

**Figure 6.**
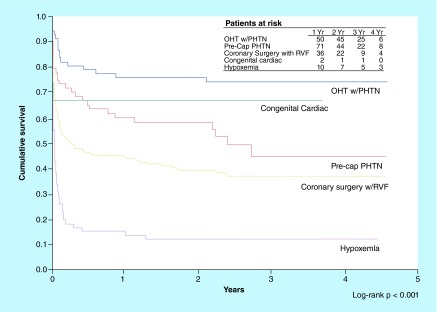
**Kaplan–Meier survival curves for inhaled nitric oxide therapy over 4 year follow-up period by indication for inhaled nitric oxide use.** Survival for OHT and OLT patients treated for pulmonary hypertension was far greater than survival in medical patients treated for life-threatening hypoxemia (p = 0.001). OHT: Orthotopic heart transplantation; OLT: Orthotopic lung transplantation; PHTN: Pulmonary hypertension; Pre-cap: Precapillary; RVF: Right ventricular failure; VAD: Ventricular assist device. Reprinted with permission from [28] © Elsevier (2006).

**Figure 7.**
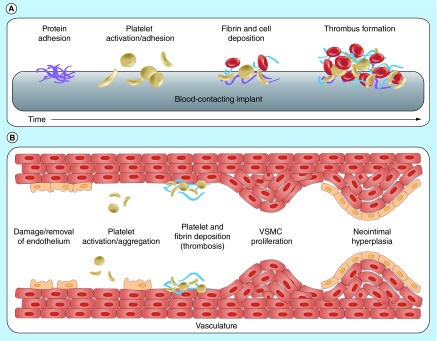
**Vascular procedures damage the endothelial lining leading to decreased production of nitric oxide required for vascular homeostasis.** This results in thrombus formation and neointimal hyperplasia with subsequent stenosis. VSMC: Vascular smooth muscle cell. Reproduced with permission from [34] © The Royal Society of Chemistry (2012).

## Nitric oxide & wound healing

The wound healing cascade is a highly complex series of events that if compromised contributes to significant morbidity in postsurgical patients. While the exact mechanism by which NO modifies wound healing remains unclear, its established antimicrobial, vasodilatory, angiogenic and collagen stimulating properties indicate the instrumental role of NO in modifying the factors essential for proper healing [1–3]. There is considerable evidence for the relationship between NO and wound healing:

States of NO deficiency, such as diabetes, malnutrition and steroid use are associated with delayed wound healing, providing clinical evidence of its importance [4];
*In vivo* studies involving inducible nitric oxide synthase (iNOS) knockout mice and those treated with iNOS inhibitors displayed delayed wound re-epithelialization and closure and decreased collagen deposition and tensile strength in the absence of NO [5];
Supplementation with arginine, an NO precursor, improved wound healing in rat models with subsequent iNOS inhibitor administration eliminating the beneficial response [6];
NO end products, nitrate and nitrite, are found in wound fluids and urinary nitrate levels remain elevated until wound closure.

In contrast to the basal production of NO by endothelial NOS (eNOS), generation of NO by iNOS in response to the inflammatory wound milieu leads to a larger, more sustained release (Figure 1). This allows NO to be effective in all three stages of the healing process – the inflammatory, proliferative and remodeling phases [7]. Macrophages, acting in the beginning of the healing cascade, are responsible for the largest production of NO and utilize NO's oxidative properties to generate reactive oxygen species for the destruction of pathogens. However, as wound healing progresses, fibroblasts, keratinocytes, endothelial cells and melanocytes take over its production, allowing NO to act continuously and impact on re-epithelialization and collagen deposition [8]. To better appreciate the multifaceted role of NO, the three overlapping yet distinctive phases of healing will be reviewed with an emphasis on how NO mediates between each stage [1]. Other sources provide a more comprehensive review of this topic [7].

The first event in the **inflammatory phase** is the formation of a fibrin plug, which maintains hemostasis and serves as a scaffold and chemo-attractant for infiltrating inflammatory cells. NO regulates vascular permeability and dilation necessary for increased trafficking of inflammatory cells to the injured site. Neutrophils and monocytes are the first on scene and initiate the removal of cellular debris and antimicrobial activity. Monocytes transform on site into macrophages, which release large quantities of NO that is then oxidized to the more toxic peroxynitrite and hydroxyl radicals. NO expression peaks within the first 1–5 days of cutaneous wound healing, correlating with the large surge of NO release by macrophages early in wound repair (Figure 2). This is confirmed by elevated levels of nitrite and citrulline, NO end products, in wound fluid as early as day 1 [7].

NO's broad spectrum antimicrobial activity helps clear the injured area of debris and facilitates the initiation of the **proliferative phase**, characterized by the laying down of new cells. Keratinocytes are stimulated to proliferate and migrate from the wound edge by macrophages that release IL-6 and NO. Angiogenesis is mediated by keratinocytes, which are the major source of VEGF-induced capillary formation. In response to hypoxia, these endothelial cells release NO, which drives increased VEGF production and protects the tissue from ischemia-reperfusion injury subsequent to increased blood flow. This new vessel formation is critical for the provisional matrix composed of collagen III, glycosaminoglycans and fibronectin laid down by proliferating fibroblasts. NO has a clear role in collagen deposition, underscored by the impact of NO donors, l-arginine and iNOS gene transfer on collagen content and its decrease in the presence of NO inhibition [1].

The disorganized interim matrix undergoes transformation in the **remodeling phase**, forming a well-organized strong network via the action of matrix metalloproteinases. The replacement of collagen III with collagen I increases the tensile strength of the tissue, with continual remodeling for up to a year.

## Utilizing NO to accelerate wound healing

NO's dual antiseptic and wound healing properties make it an ideal agent to treat chronic, nonhealing wounds. However, its high reactivity and short half-life makes therapeutic design a challenge. Different platforms of NO delivery have been evaluated, with promising investigative data presented here. For a more comprehensive review of NO donors and their mechanism of NO release, refer to [10].

Gaseous nitrate (gNO) administered directly to a chronic, nonhealing leg ulcer via a gas-diluting delivery system resulted in 90% wound closure by 6 weeks after a 14 day treatment regimen [11]. Despite positive results, use of this therapy in a setting outside of a specialty clinic is complicated due to the expense and obligatory delivery from a gas tank. In addition, the difficulty of excluding oxygen to prevent NO's transformation to NO_2_, makes this treatment regimen cumbersome and potentially toxic to host cells;
Acidified nitrate creams generate NO via the reduction of nitrite to NO via ascorbic acid. Topical application was found to accelerate wound healing in normal and diabetic mice as measured by percent wound closure and wound half closure time [12]. The ease of administration makes this agent a likeable option for targeted NO delivery. However, it has been shown to cause cutaneous inflammation and erythema at the site of application, limiting its use;
Diazeniumdiolates (or NONOates) are of particular interest in the study of NO for wound healing due to the simplicity of synthesis and its predictable, controlled release of NO in aqueous environments. Synthetic NO donors are formed via the reaction between NO and a variety of different amine groups. A polyethyleneimine cellulose NONOate polymer (PEIC-NO) applied topically to full thickness dermal wounds of rats accelerated closure and increased urinary nitrite excretion [13]. In addition, a polyvinyl NONO-ate hydrogel wound dressing cultured with human dermal fibroblasts induced a significant increase in collagen production. Preliminary studies with these hydrogel platforms in diabetic mice showed thicker granulation and scar tissue in the treatment arms but no statistically significant increase in time to wound closure [14]. Despite its advantages, not all NONOates are clinically useful due to the potential of carcinogenic nitrosamine formation through interactions between NONOate decomposition products (e.g., V-PYRRO/NO metabolism yields the hepatocarcinogen, *N*-nitrosopyrrolidine). In addition, water soluble NONOates are limited by migration from the wound prior to NO release and, as such, further studies should focus on water-insoluble forms to circumvent this problem;
Probiotic NO-releasing patches utilize the anaerobic metabolism of bacteria to deliver gNO topically. Immobilized *Lactobacilli* ferment glucose into lactic acid, which then reacts with sodium nitrite to produce gNO that can diffuse freely through the adhesive. This platform was shown to accelerate healing in ischemic wounds of New Zealand's white rabbit models [15], and may provide a safe and cost effective treatment of chronic wounds;
Topical delivery of NO nanoparticles (NO-np) in murine models led to faster wound closure, as determined by histological examination revealing increased macrophage recruitment, vascularization and collagen deposition in the treated group (Figure 3) [16]. NO-nps were also effective in an immunocompromised model, accelerating healing in diabetic, severe combined immunodeficient mice compared with control and those treated with analog concentrations of DETA-NONOates [17]. Nanoparticles are an emerging area of investigation as its unique composition allows for sustained and controlled release, with minimization of side effects due to enhanced targeting. Its advantageous safety profile overcomes one of the major challenges of NO use.

## Nitric oxide & ischemia-reperfusion injury

Ischemia-reperfusion (IR) injury is a perioperative complication of any procedure that restricts blood flow to a particular organ followed by subsequent reperfusion and reoxygenation. Although treatment of tissue hypoxia would intuitively involve reintroduction of oxygen, restoration of blood flow often induces a profound inflammatory response characterized by neutrophilic infiltration and microcirculatory flow disturbances [18,19]. IR injury is of particular importance in solid-organ transplantation as it is a known contributor of early-graft failure and acute and chronic rejection [20]. Ischemia occurs during organ preservation (cold ischemia) and when blood flow to recipient tissue is blocked (warm ischemia), with reperfusion injury occurring upon reanastomosis.

In early ischemia, hypoxic conditions induce endothelial production of NO via eNOS. The rapid accumulation of NO depletes the available pool of l-arginine, which triggers the uncoupling of eNOS and a subsequent decrease in NO generation. Inadequate NO levels make tissue more susceptible to injury as NO serves many protective functions [21,22]:

Anti-oxidant: NO acts as an oxygen radical scavenger and modulates cellular respiration by competing with oxygen for binding to cytochrome-c oxidase on the electron transport chain. In oxygen-deficient states NO binding is more pronounced, creating a preconditioning state that protects against the generation of reactive oxygen species after reperfusion and the resumption of the electron transport chain (Figure 4) [23];
Anti-adhesive: NO inhibits the initial attachment and adherence of leukocytes to the endothelium via the prevention of selectin expression;
Anti-inflammatory: NO reduces the release of TNF-α and IL-1, proinflammatory cytokines that trigger neutrophil accumulation;
Anti-apoptotic: NO protects against apoptosis via the downregulation of P53 and the activation of heat-shock proteins. In addition, *S-*nitrosylation of proteins modifies intracellular handling of calcium, leading to reduced cell apoptosis.

The protective properties of NO are concentration dependent and adverse effects can occur with excess systemic administration. Additionally, since the inflammatory cascade in IR injury varies between organs, targeted delivery of NO to compromised tissue is a promising new avenue of investigation. In one study, *S-*nitrosothiols were targeted to hepatic cells via incorporation of mannose and galactose ligands that specifically bind the mannose receptor and asialoglycoprotein receptor, respectively, on hepatic cells. This resulted in suppression of alanine and aspartate aminotransferase levels, inhibition of NF-κB activation and decrease in neutrophil population due to significantly greater concentrations of NO macromolecules in liver as compared to circulating blood [25]. In another study, infusion of mitochondria-targeted *S-*nitrosothiols during reperfusion of the heart mitigated IR injury, likely due to the persistent *S*-nitrosation of complex I and other mitochondrial proteins [26].

A recent systematic review of NO supplementation in human subjects outlined the variable success of different NO donors for organ transplantation, cardiopulmonary bypass, myocardial infarction and limb tourniquet studies [23]. The preponderance of preclinical animal data and human trials suggests that NO administration can successfully mitigate IR injury. However, given the heterogeneity of donors used and disease states studied, further investigation is still necessary to quantitatively confirm this current hypothesis.

## Perioperative properties of inhaled NO

Inhaled NO (iNO) has been investigated for the perioperative treatment of ischemia-reperfusion injury. It was initially hypothesized that upon reaching the bloodstream, NO would be rapidly scavenged by hemoglobin and have no effect systemically. However, it has been shown that in the pulmonary vasculature some NO is converted to stable NO donors (e.g., nitrite, *S-*nitrosothiols) that can regenerate in the periphery and impact platelet aggregation and neutrophil migration (Figure 5) [27].

However, more important than its protective role in IR injury, iNO's greatest significance is as a selective pulmonary vasodilator. iNO reduces pulmonary vascular resistance without affecting arterial circulation and has therefore been harnessed for the perioperative treatment of pulmonary hypertension [30]. Indications for its use in surgery include:

Congenital and adult cardiac surgery;
Orthotopic lung transplantation;
Orthotopic heart transplantation;
Ventricular device assist placement.

A retrospective study investigating the long-term outcomes of iNO therapy showed that iNO has greatest benefit for transplant patients (Figure 6) [28]. Many patients undergoing cardiac transplantation have standing pulmonary hypertension secondary to years of congestive heart failure, exacerbated intraoperatively by cardiopulmonary bypass and blood transfusions [29]. The right ventricle of the allograft is not well adapted to meet increased afterload strain due to cold ischemia and coronary-artery distribution, which precludes optimal-ventricular perfusion. Use of iNO was found to alleviate the afterload and has even been used to screen patients eligible for surgery [29].

## Nitric oxide & vascular surgery

Vascular procedures invariably manipulate and damage the endothelial-cell monolayer, leading to disrupted production of NO and loss of its vasoprotective properties. NO is responsible for the maintenance of a thromboresistant endothelial lining and decreased levels results in platelet aggregation, inflammatory-cell infiltration and vascular smooth muscle proliferation which threatens vessel patency [31,32]. Surgical placement of implantable and extracorporeal blood-contacting devices are frequently complicated by functional failure due to the thrombogenic nature of synthetic surfaces (Figure 7) [33]. Many studies have attempted coating devices with endothelial cells as a means of reintroducing NO. However, transplanted endothelial cells behave differently than normal cells and do not produce biologically effective concentrations of NO. Systemic administration of NO agents via iNO or NO-donating drugs (NO-aspirin hybrid, linsidomine) has not proven efficacious either as biologically ineffective doses are required to avoid toxic adverse events [32].

Researchers have moved toward the incorporation of NO-donating drugs, particularly diazeniumdiolates and *S-*nitrosothiols, into hydrogels and polymers as a means of generating NO locally for extended periods of time [31,32]. These materials are applied directly to the vessel injury, which avoids loss of NO by hemoglobin scavenging:

Hydrogels can be used in both open (applied to outside of vessel) and endovascular (coating the angioplasty balloon) procedures, and allows for high NO loading and uniform diffusion of drug into the arterial wall with minimal loss into the bloodstream. However, they have to be made at the time of surgery, which limits their use. In one study, a polylactic-polyglycolic acid polymeric matrix containing 2.5% SPER/NO applied to rat balloon injured arteries significantly suppressed injury-induced activation of NFκβ and neointimal hyperplasia [35]. In a similar model, *S-*nitrosocysteine (Cys-NO) hydrogels were found to release drug contents for 24 h and inhibited smooth-muscle proliferation and platelet adherence [36];
Polymers can be stably stored and incorporated into vascular grafts and stents to release definite amounts of NO over an extended time period. Diazeniumdiolated silica nanoparticles embedded into hydrophobic matrices were used to coat the inside of blood-circulatory tubes in a rabbit model, which resulted in less platelet consumption and activation when compared with controls [37]. Nascent research focuses on using endogenous, circulating *S-*nitrosothiols, which are not limited by a finite amount of drug. An l-cysteine incorporated polymer was shown to transnitrosate NO from AlbSNO to the immobilized l-cysteine, leading to 65% reduction of platelet adherence *in vitro* [38].

Echogenic liposomes loaded with gaseous NO have been evaluated as a method of site-specific NO delivery that does not require direct vessel application [39]. Triggerable NO release by Doppler ultrasound in injured rabbit carotid arteries resulted in significantly reduced neointimal hyperplasia, with a sevenfold increase in NO delivery compared with NO-saturated solutions. This method of encapsulation allows for the utilization of gaseous NO while avoiding its caustic side effects.

The above-mentioned methods are still limited by the delivery of a finite quantity of drug and future directions focus on harnessing endogenous *S-*nitrosothiols circulating in blood as a continuous source of NO. Due to its great clinical import, the utilization of NO in the realm of vascular-biomedical engineering remains an active and ongoing area of research and translation of these materials into clinical studies is on the horizon.

## Nitric oxide & orthopedics

Bone is a complex living tissue in a constant state of dynamic turnover carried out by osteoblasts that deposit new bone and osteoclasts that break down the existing matrix [9]. Despite the impressive regenerative potential of bone, invasive orthopedic procedures result in impaired tissue healing and infection, particularly for open-fracture and joint-revision surgeries. [2] Nitric oxide contributes to the remodeling process by directing osteoblast and osteoclast activity, which in addition to its antimicrobial and wound healing properties, makes it an innovative agent for localized therapeutics. Generated by constitutive expression of eNOS, NO directs normal bone remodeling and responds to estrogen stimulation and mechanical strain by promoting osteoblast proliferation and differentiation. NO is also involved in bone repair exhibited by the increased expression of all three NOS isoforms following fracture. In addition, administration of l-name, a nonspecific NOS inhibitor, and deletion of the iNOS gene are seen to delay fracture healing. NO-releasing scaffolds have been designed to allow for on-site action of NO to accelerate tissue repair. One study investigating the effects of a demineralized bone matrix solution with *S-*nitrosobovine serum albumin (SNO-BSA) on fracture healing found increased union across bone defects, and enhancement in bone-mineral density and cortex modeling in the SNO-BSA group. [40] In another study, a surgically implanted NO-releasing chitosan matrix at the fracture site resulted in 20% increase in cross-sectional area of the fracture callus. [41] The antimicrobial properties of NO have been harnessed to prevent postoperative infection by coating external fixation pins with diazeniumdiolate NO donor-functionalized xerogels. This reduced the incidence of infection in the treatment arm as compared with controls, and the surrounding surgical wounds showed qualitatively decreased erythema and edema. [42] Infected implants complicate both bone and adjacent tissue healing and form a nidus for recurring infection that necessitates removal. The use of NO for orthopedics is still in the nascent stages of investigation and more research in this area is needed for the clinical translation of NO for postoperative recovery.

## Conclusion

Nitric oxide has broad anti-inflammatory and anti-thrombotic properties that can be harnessed for the treatment and prevention of surgical complications, particulary related to wound repair, ischemia reperfusion injury and graft failure/infection. However, while NO in theory has tremendous therapeutic potential, the greatest impediment to clinical translation is site-specific delivery. Further research into methods of localized treatment is required for meaningful application of NO in the clinical realm.

**Executive summary**

Nitric oxide's (NO) role in angiogenesis, endothelial homeostasis and inflammation makes it a significant player in the host response to surgery, mitigating-wound healing and ischemia-reperfusion injury.
Wound healing occurs in three overlapping, yet distinct phases – inflammatory, proliferative and remodeling, with endogenous production of NO accelerating the process.
In the early stages of healing, a surge of NO production by macrophages allows for the removal of cellular debris and pathogens, while in the later stages, NO mediates angiogenesis, keratinocyte proliferation and collagen deposition.
Exogenous delivery of NO to cutaneous wounds has been shown to enhance wound healing, prompting investigation into novel topical platforms, including hydrogel dressings and nanoparticles.
NO alleviates ischemia-reperfusion injury, a common complication of surgical procedures, particularly organ transplantation.
Inhaled NO is being investigated as a prophylactic and therapeutic for ischemia-reperfusion injury as well as an adjuvant for perioperative pulmonary hypertension.
Damage to the vasculature during interventional procedure leads to the loss of endothelial-derived NO, and its thromboresistant properties, contributing to the high rate of post procedure restenosis and graft failure.
NO's antimicrobial and bone remodeling features are being harnessed to transform the postoperative recovery of orthopedic patients.
